# Long‐term monitoring of tropical alpine habitat change, Andean anurans, and chytrid fungus in the Cordillera Vilcanota, Peru: Results from a decade of study

**DOI:** 10.1002/ece3.2779

**Published:** 2017-02-07

**Authors:** Tracie A. Seimon, Anton Seimon, Karina Yager, Kelsey Reider, Amanda Delgado, Preston Sowell, Alfredo Tupayachi, Bronwen Konecky, Denise McAloose, Stephan Halloy

**Affiliations:** ^1^ Wildlife Conservation SocietyZoological Health ProgramBronxNYUSA; ^2^ Department of Geography and PlanningAppalachian State UniversityBooneNCUSA; ^3^ School of Marine and Atmospheric SciencesStony Brook UniversityStony BrookNYUSA; ^4^ Department of Biological SciencesFlorida International UniversityMiamiFLUSA; ^5^ Museo de Historia NaturalUniversidad Nacional de San Antonio Abad del CuscoCuscoPeru; ^6^ Ausangate Environmental LLCBoulderCOUSA; ^7^ Universidad Nacional de San Antonio Abad del CuscoHerbarioCuscoPeru; ^8^ Cooperative Institute for Research in Environmental SciencesUniversity of Colorado BoulderBoulderCOUSA; ^9^ Ministry for Primary IndustriesWellingtonNew Zealand

**Keywords:** amphibian decline, chytridiomycosis, climate change, deglaciation, ecological succession, highest amphibians, *Telmatobius*, tropical Andes

## Abstract

The Cordillera Vilcanota in southern Peru is the second largest glacierized range in the tropics and home to one of the largest high‐alpine lakes, Sibinacocha (4,860 m). Here, *Telmatobius marmoratus* (marbled water frog), *Rhinella spinulosa* (Andean toad), and *Pleurodema marmoratum* (marbled four‐eyed frog) have expanded their range vertically within the past century to inhabit newly formed ponds created by ongoing deglaciation. These anuran populations, geographically among the highest (5,200–5,400 m) recorded globally, are being impacted by the chytrid fungus *Batrachochytrium dendrobatidis* (*Bd*), and the disease it causes, chytridiomycosis. In this study, we report results from over a decade of monitoring these three anuran species, their habitat, and *Bd* infection status. Our observations reveal dynamic changes in habitat including ongoing rapid deglaciation (18.4 m/year widening of a corridor between retreating glaciers from 2005 to 2015), new pond formation, changes in vegetation in amphibian habitat, and widespread occurrence of *Bd* in amphibians in seven sites. Three of these sites have tested positive for *Bd* over a 9‐ to 12‐year period. In addition, we observed a widespread reduction in *T. marmoratus* encounters in the Vilcanota in 2008, 2009, and 2012, while encounters increased in 2013 and 2015. Despite the rapid and dynamic changes in habitat under a warming climate, continued presence of *Bd* in the environment for over a decade, and a reduction in one of three anuran species, we document that these anurans continue to breed and survive in this high Andean environment. High variability in anuran encounters across sites and plasticity in these populations across habitats, sites, and years are all factors that could favor repopulation postdecline. Preserving the connectivity of wetlands in the Cordillera Vilcanota is therefore essential in ensuring that anurans continue to breed and adapt as climate change continues to reshape the environment.

## 1 Introduction

High‐alpine ecosystems (Körner, 2003) are highly sensitive to climatic perturbations and provide opportunities to study how species adapt to rapidly changing environments. In the Cordillera Vilcanota, Peru, the second largest glacierized range in the tropics, rapid deglaciation promoted a 150–200 m vertical range extension of amphibians within the last century (Seimon et al., 2007). Here, three species of amphibians have colonized a recently deglaciated corridor at the highest elevations—5,200–5,400 meters above sea level (asl; all elevations hereafter are asl)—recorded for amphibian species (Seimon & Seimon, 2015; Seimon et al., 2007). These observations parallel those from the Tsaratanana Massif in Madagascar, where upslope shifts of 19–51 m over a decade in 30 species of amphibians have been reported (Raxworthy et al., 2008).

The amphibian chytrid fungus *Batrachochytrium dendrobatidis* (*Bd*) has been detected in amphibians from several sites in the Cordillera Vilcanota (Seimon et al., 2007). *Bd* is an amphibian pathogen that has become widespread throughout Peru and can cause the disease chytridiomycosis (Catenazzi, Lehr, Rodriguez, & Vredenburg, 2011; Catenazzi, Lehr, & Vredenburg, 2014; Catenazzi, Vredenburg, & Lehr, 2010; Catenazzi & von May, 2014;Kosch, Morales, & Summers, 2012; von May et al., 2008). Infection by this waterborne fungus—which colonizes skin—compromises epidermal respiration, thermoregulation, and hydration and ultimately can kill its host (Voyles et al., 2009). In South America, declines in a number of species including high Andean genera such as *Telmatobius* have been attributed to chytridiomycosis: Declines in at least ten species of *Telmatobius* from Ecuador, Peru, and Argentina have been attributed to or associated with *Bd* (Barrionuevo & Mangione, 2006; Barrionuevo & Ponssa, 2008; Catenazzi & von May, 2014; Catenazzi, von May, & Vredenburg, 2013; Catenazzi et al., 2011, 2014; Merino‐Viteri, Coloma, & Almendariz, 2005; Seimon, Hoernig, Sowell, Halloy, & Seimon, 2005; Seimon et al., 2007; von May et al., 2008), and several studies have documented chytridiomycosis in this genus (Barrionuevo & Mangione, 2006; Ron & Merino, 2000; Seimon et al., 2005, 2007). *Bd* has been documented in *Telmatobius marmoratus* and the Critically Endangered *Telmatobius culeus* (Titicaca water frog) from Lake Titicaca, Bolivia (Berenguel, Elias, Weaver, & Reading, 2016; Cossel, Lindquist, Craig, & Luthman, 2014; Zevallos, Elias, Berenguel, Weaver, & Reading, 2016), and in captive *T. marmoratus* in markets of Peru, indicating that this pathogen is also being moved around by the food trade (Angulo, 2008; Catenazzi et al., 2010; Kosch et al., 2012; von May et al., 2008).

*Telmatobius* are found in high mountain streams and lakes throughout the Andes, and many species from this genus are rapidly disappearing (Angulo, 2008; von May et al., 2008). According to the IUCN Red List of Threatened Species, 33 of 59 (56%) *Telmatobius* species are classified as Endangered or Critically Endangered, 45 (76%) are Threatened, and 41 species (69%) have populations that are decreasing (http://www.iucnredlist.org; accessed June 29, 2015). Five threats have been identified as having an important role in *Telmatobius* declines: habitat degradation and loss; overharvesting for food consumption; introduced predators including trout; pollution; and infectious diseases, notably chytridiomycosis and ranavirus (Angulo, 2008; Warne, LaBumbard, LaGrange, Vredenburg, & Catenazzi, 2016).

Our long‐term study was prompted in 2002 by anecdotal observations, related by indigenous Quechua herders living above 3,800 m, that amphibians were disappearing throughout the Cordillera Vilcanota (Seimon et al., 2005, 2007). Subsequent field surveys supported their observations. In 2002 and 2003, we identified two amphibian species, *T. marmoratus* (marbled water frog) and *Pleurodema marmoratum* (marbled four‐eyed frog), exhibiting the clinical symptoms of and/or the disease chytridiomycosis (Seimon et al., 2005, 2007). Then, in 2004, a mortality event occurred shortly before a site survey, in which 24 adult and juvenile *T. marmoratus* were found dead, with evidence of shedding skin, in a pond where *Bd* had been identified the previous year (Seimon et al., 2007); *Bd* was implicated in the die‐off. Following these findings, our working hypothesis guiding the subsequent investigations reported here was that continued presence of the invasive pathogen *Bd* would promote continued declines in anuran populations, possibly culminating in local extinctions.

The Cordillera Vilcanota region, including Lake Sibinacocha, is a long‐term climate change monitoring site of the GLORIA network (Cuesta et al., 2012; Halloy et al., 2010), and this site has recently been recommended as a high‐priority area for long‐term studies of amphibian populations in Peru (Catenazzi & von May, 2014). The area of the sampling sites spans 16 by 25 km, with a 1,038‐m elevational difference (4,362–5,400 m) between lowest and highest sites. At such elevations, atmospheric pressure is 50%–60% that of sea level, and plants are adapted to a lower CO_2_ environment (Halloy, 1981). Global elevational records have been documented across our field region for agricultural (potato) and flowering plants, orchids, amphibians, pelecypods, and lizards (Seimon et al., 2007). At such elevations, animals and plants develop unique adaptations: Lizards become viviparous, amphibians can call and mate under ice, and both lizards and amphibians can freeze at night and revive to a normal active state the next morning (Halloy, 1989; Halloy et al., 2010). Here, we present results from monitoring habitat change, population trends, and the presence of *Bd* for three anuran species—*T. marmoratus*,* P. marmoratum*, and *Rhinella spinulosa*—in this region over the past decade.

## 2 Materials and Methods

### 2.1 Ethics statement

All work complied with guidelines for the use of live amphibians in field research by the American Society of Ichthyologists and Herpetologists, the Society for the Study of Amphibians and Reptiles, and the Herpetologists' League.

Individual frogs were shaded from the sun during handling, monitored for signs of stress, and replaced into the location in which they were found. Recommended biosecurity practices for minimizing risk of disease transmission between animals and field sites were followed at all sites (Pessier & Mendelson, 2009). Field surveys were conducted in accordance with the Declining Amphibian Population Task Force Fieldwork Code of Practice (http://www.amphibianark.org/pdf/Husbandry/The%20DAPTF%20Fieldwork%20Code%20of%20Practice.pdf) and Brem, Mendelson, and Lips (2007).

### 2.2 Field surveys, animal handling, sample collection, storage

**Figure 1:**
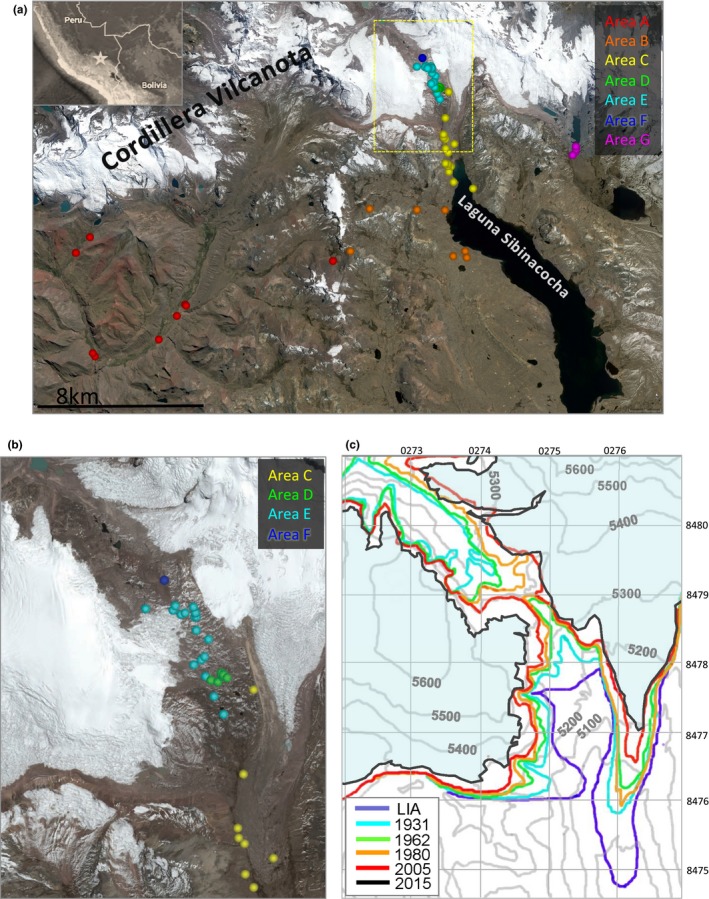
(a) Satellite map from September 2013 showing locations of individual survey sites in areas A–G. Star on inset map locates field region in broader geographic context, and yellow box indicates area of interest shown in (b). (b) Zoomed subsection showing sites in areas D–F. (c) Topographic map section (100‐m contour interval) of the pass region in (b), and the glacial margin positions at the ca. 1850–1900 Little Ice Age maximum (LIA, purple), 1931 (cyan), 1962 (green), 1980 (gold), 2005 (red), and 2015 (black). Glacial coverage in 2015 is shaded light blue. 1‐km UTM grid is overlaid for reference

Nine research expeditions of 5‐ to 14‐day duration were conducted between 2003 and 2015 (for dates, see Tables S2–S3). The 16 × 25 km survey area is located ~100 km ESE of Cusco, Peru, in the Cordillera Vilcanota, and focused in the Lake Sibinacocha watershed (13.85°S, 71.05°W; 4,860–6,100 m). Between 2002 and 2015, we established and visited 63 individual sites and three elevational transects, all clustered within seven areas (denoted as areas A–G), across a broad elevational range (4,362–5,400 m) (Figure 1a and Table S1). Areas share characteristics of connected watercourses within a major water catchment. Areas A–F were established between 2002 and 2005 (Seimon et al., 2007); Area G was added in 2015. Not all sites and areas were sampled during each field expedition.

Three 5 × 300 m herpetological transects were developed as part of the Global Observation Research Initiative in Alpine Environments (GLORIA) network (Pauli et al., 2015), following the GLORIA herpetological observation protocol (Seimon, Seimon, Halloy, & Musicante, 2015). The direction of each transect was chosen based on the availability of amphibian habitat and water sources extending down 300 m from the summit anchor. Two transects are located in Area E, and the third is in Area B. During site visits, two to four researchers performed visual encounter surveys, obtained GPS coordinates, and took photographs.

Locations for each survey site were recorded using the Provisional South America 1956 datum, for consistency with the Peruvian *Instituto Geografico Nacional* topographic map series, and are provided in Table S1. Researchers surveyed each site by carefully lifting rocks and sifting ponds with nets to locate tadpole, juvenile, and adult anurans and recorded vegetation types (aquatic and terrestrial). Captured animals were examined for signs of illness, measured for snout‐to‐vent length, photographed, swabbed, and returned to the site of capture where they were then released.

Skin swabs were collected using BBL 1/8″‐diameter sterile rayon‐tipped swabs (Fisher Scientific, Pittsburgh, PA, USA). Each frog was captured in a clean, unused plastic bag and swabbed four to five times each on the underside of the hind feet, thighs, abdomen, and forefeet, using moderate pressure to ensure collection of skin tissue without causing injury to the animal. Tadpoles were swabbed by gently rotating the swab across the mouthparts for five turns. Tadpoles from the same pond, or frogs found sharing a burrow under the same rock, were placed together in the same bag; otherwise, frogs were placed in separate bags. Swab samples were air‐dried and stored in airtight plastic containers at ambient temperature. Handlers changed powderless, nitrile gloves between each animal sampled to avoid potential cross‐contamination. Footwear, nets, rulers, and instruments were sprayed with ethanol (90% solution) between sites.

### 2.3 Ice margin mapping

The generation of the map demonstrating temporal evolution of glacial recession has been previously described (Seimon et al., 2007). This map was updated to add a new isoline using a satellite image obtained from ImageHunter by Apollo Mapping (Boulder CO: catalog ID *103001004AC52300* and *1030010049008200*, sensor WV2, resolution, 50 cm, acquisition date, 13 October 2015). The image was rescaled to the base map projection. The brightness gradient of the ice margin was then used to define the isoline. Distances were calculated and map overlays created using GPS coordinates from field observations and Google Earth Pro and Google Maps online software.

### 2.4 Temperature recording

A HOBO Tidbit V2 temperature datalogger (#1246644) was deployed at 30 cm water depth at Area D Pond 5 on 14 March 2008 and retrieved on 2 August 2009. A second datalogger, installed and collected on the same dates, was placed in a ventilated and shaded rock cavity on a hill summit 150 m NE of D5 Pond at 5,250 m.

### 2.5 Climatological observations

Meteorological records were used to assess seasonal character leading up to site visits and identify conditions relative to comparable time‐of‐year averages. Long‐term climatological observations are not available from within the Sibinacocha watershed, so to characterize climatic variability and trends, we used 15‐year daily precipitation and temperature data series from nearby weather stations to serve as proxies for climatological conditions in the watershed; the data are from 1 July 2000 to 30 June 2015. The stations, operated by the Peruvian *Servicio Nacional de Meteorologia y Hidrologia* (SENAMHI)—at Sicuani (3,574 m), 57 km to the south; Ccatcca (3,729 m), 54 km west‐northwest; and Macusani (4,345 m), 78 km east‐southeast of Area D, have similar annual precipitation means (732, 709, and 664 mm, respectively). Daily temperature maxima and 24‐hr precipitation totals from the three sites were averaged and then assessed as seasonal (90‐day) moving averages. Unlike daytime maxima, nocturnal minimum temperatures exhibit large variations dependent upon local environmental factors so were not used for the analysis.

### 2.6 Polymerase chain reaction testing

Samples collected for *Bd* testing were air‐dried and frozen until analysis in 2013 and 2015. For *Bd* analysis, DNA was extracted from the swabs using 150 μl of PrepMan (Life Technologies, Grand Island, NY, USA) and extracts were diluted 1:10 in RNase/DNase‐free water. The samples were analyzed in singlicate by real‐time quantitative polymerase chain reaction (PCR) amplification of the internal transcribed spacer (ITS‐1) and 5.8S rDNA region using established methods (Boyle, Boyle, Olsen, Morgan, & Hyatt, 2004). Positive samples were retested in triplicate. TaqMan PCR assays were conducted using a Bio‐Rad Mini‐Opticon Real‐Time PCR detection system. Twenty‐microliter reactions containing 10 μl of 2× TaqMan Environmental Master Mix (Life Technologies), 900 nmol/L of each primer (ITS‐1 Chytr3 and 5.8S Chytr), 250 nmol/L of Chytr MGB probe (Life Technologies), 2.5 μl of 10× exogenous internal positive control primers and probe, 0.5 μl of 50× exogenous internal positive control DNA (TaqMan^®^ Exogenous Internal Positive Control kit; Life Technologies), DNase/RNase‐free water, and 5 μl of diluted DNA were added to each well of a 48‐well plate. The exogenous internal positive control reagents served as inhibition controls in the PCR reactions. PCR amplification was run as previously described (Seimon, Ayebare, et al., 2015). Purified genomic *Bd* DNA or *Bd* plasmid carrying the ITS1‐5.8S‐ITS2 region was provided by Dr. Allan Pessier (San Diego Institute for Conservation Research, CA, USA) and was diluted to a range of concentrations to generate a standard curve. The copy number was calculated as described previously (Seimon, Ayebare, et al., 2015).

## 3 Results

**Figure 2:**
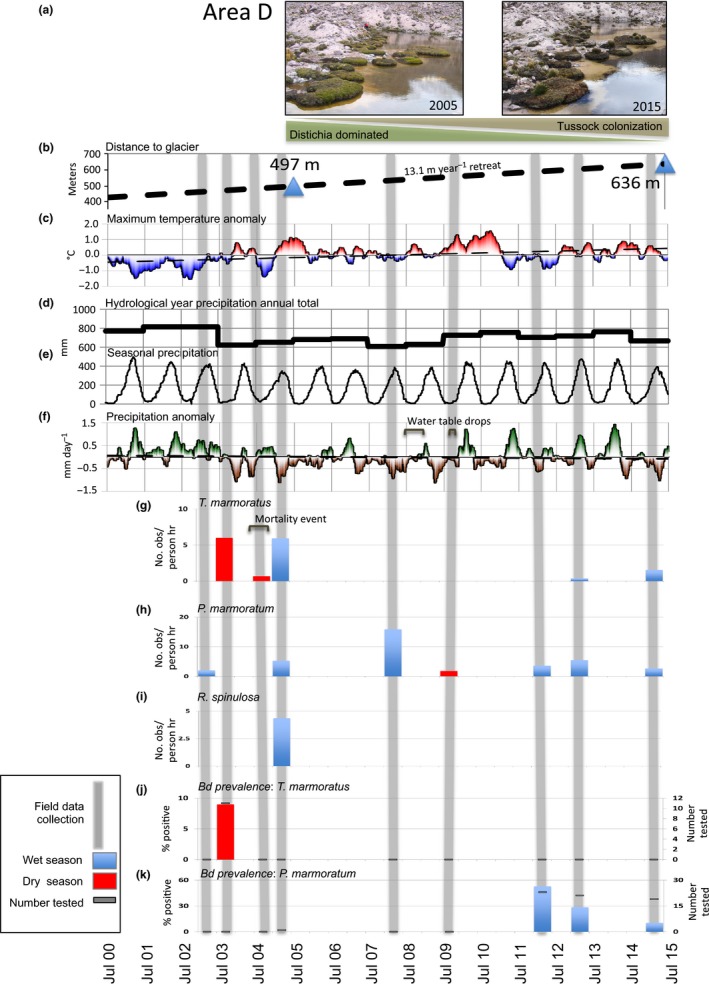
Fifteen‐year timeline of observational data between July 2000 and June 2015 comparing environmental and climatological parameters to amphibian data from Area D. Grey vertical bars indicate times of field expeditions. Shown from top to bottom: (a) repeat images of *Distichia muscoides* (green cushion plants) surrounding D1 Pond in Area D in March 2005 and March 2015. (b) Distance (m) from Area D1 Pond to closest ice margin in 2005 and 2015, and the rate of deglaciation between these two points. (c) Seasonal temperature anomalies for daily maximum temperature (°C, 90‐day running mean) relative to 15‐yr daily means; dashed line shows linear trend over the time period. (d) Hydrological year (July–June) precipitation totals and (e) seasonal precipitation sum (90‐day running total), both in mm. (f) Seasonal precipitation anomalies (mm/day, 90‐day departure from mean) relative to 2000–2015 daily mean rates. Comparison of *Telmatobius marmoratus* (g)‐, *Rhinella spinulosa* (h)‐, and *Pleurodema marmoratum* (i)‐adjusted encounters (adult, juvenile, and tadpole combined) recorded during the dry seasons (red) of 2003, 2004, and 2009, and the wet seasons (blue) of 2003, 2005, 2008, 2012, 2013, and 2015 in Area D. The percent of samples that tested positive in the wet season (blue) and dry season (red) for *Batrachochytrium dendrobatidis* for *T. marmoratus* (j) and *P. marmoratum* (k). Grey bars denote the number of samples tested

Three anuran species, *T. marmoratus*,* P. marmoratum,* and *R. spinulosa*, were intermittently monitored at seven locations (areas A–G) during visits between 2003 and 2015. Locations of all 63 individual sites are shown in Figure 1a,b and Table S1. During site visits, we documented habitat change, glacial recession, vegetation changes, and anuran encounters and monitored for the presence of *Bd*. We also examined local climatological variability and trends in postanalysis using SENAMHI station observations. A variety of these data types are displayed along a common 15‐year timeline in Figure 2 and are discussed below.

### 3.1 Habitat change

Observations from six field expeditions conducted from 2005 to 2015 demonstrate that deglaciation and associated anuran habitat change, previously reported in Seimon et al. (2007), continue to the present. Changes are most pronounced in the uppermost areas D–F in the periglacial zone, where ice margins, vegetation, and water levels continue to evolve dynamically.

#### 3.1.1 Glacial margin changes

**Figure 3:**
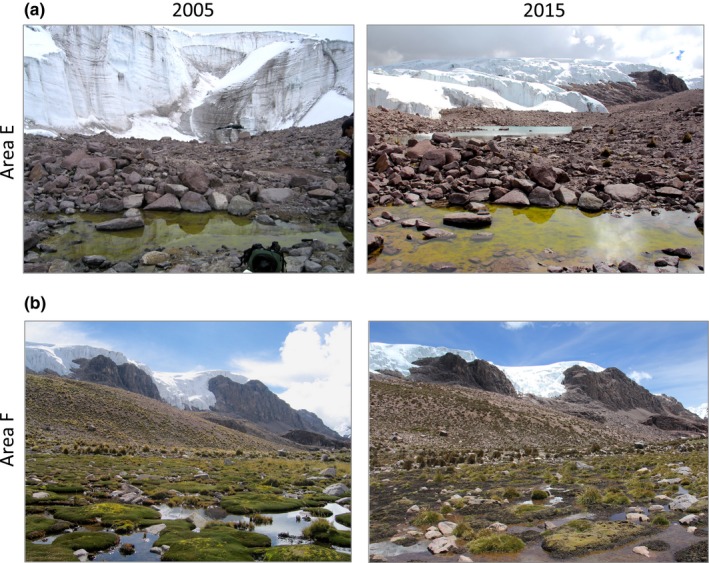
(a) Repeat image of E7 Pond in March 2005 and March 2015. The glacier was 45 m from the pond when *Pleurodema marmoratum* tadpoles were first observed in 2005; the 2015 image reveals significant ice recession and newly formed E 2008 Pond. (b) Repeat image of Area F Pond in March 2005 and March 2015. The 2005 image shows extensive *Distichia muscoides* that is flowering and appears healthy. The 2015 image shows a die‐off of the cushion plants; new tussock grasses have colonized and are growing on the peat

A decade of new observations identifies that the rapid rate of deglaciation previously reported has further accelerated. An ice margin isoline from 2015 added to the chronosequence map presented in Seimon et al. (2007) reveals significant post‐2005 glacial recession near areas D, E, and F (Figure 1c). The ice‐free corridor that first became established around 1980 had a minimum width of 238 m in March 2005 (Seimon et al., 2007) and expanded to 422 m in 2015. The average widening rate of 18.4 m/year between 2005 and 2015 is almost double the rate (9.52 m/year) reported for 1980–2005 (Seimon et al., 2007). In Area E, the deglaciated corridor E7 Pond was located 45 m from the glacial ice wall in 2005 (Figure 3a, left panel), but continued recession averaging 7.5 m/year through 2015 has transformed the local landscape markedly, including a new pond that formed in 2008 (E 2008 Pond) (Figure 3a, right panel). Tadpoles of *P. marmoratum*, first documented in E7 Pond in 2005, were also observed in 2015. The ice margin uphill from Area D receded even more rapidly, averaging 13.1 m/year (Figure 2b).

#### 3.1.2 Vegetation changes

Repeated site visits revealed ecological successions in progress during our period of observations. Contrary to the ongoing general pattern of uphill vegetation range and coverage increases observed at the GLORIA sites near Area D (Cuesta et al., 2012; Cuesta et al.), *Distichia muscoides* cushion plants on the periphery of monitored ponds exhibited signs of decline. The location and orientations of Area D ponds have been described previously (Seimon et al., 2007). In March 2005, we observed significant die‐off of *D. muscoides* in rings surrounding patches of living plants in D1 (Figure 2a, left panel), D2, and D5 ponds, with small areas of die‐off in D3 and D6 (the most downstream pond); however, the majority of individual plants contained areas that were robust and green. By March 2015, the die‐off of *D. muscoides* had progressed, and most plants had desiccated completely around D1 (Figure 2a, right panel), D2, and D5, with some die‐off noted around D3 and along the east perimeter of D6. Tussock grasses were observed growing in the remnants of the dead plants around D1, D2, D3, and D5.

When visited in 2005, the pond at Area F, which developed in 1970 (Seimon et al., 2007), appeared to support a stable cushion peatland ecosystem (also known as *bofedale*,* ciénaga*, and vascular plant peatland; Figure 3b, left panel). The cushion peatland was dominated by *D. muscoides*, tussock grasses, and perennial plants and existed as a perennial pond. When revisited in the 2008 wet season, *D. muscoides* exhibited signs of desiccation with central patchy areas of green surrounded by dead vegetation around the edges, even though there was ample water in the central pond of the cushion peatland. A dry season visit in 2009 found both the pond and cushion peatland completely dry, with wind‐blown algal mats on the dusty lakebed. An image from March 2015 (Figure 3c, right panel) shows water within the pond during the wet season, and extensive growth of tussock grasses over the *D. muscoides* peat, which had died off as this pond had apparently transitioned from being perennial to seasonally ephemeral.

#### 3.1.3 Climatic variability and trends

For indications of climatological anomalies leading up to the time of each site visit, we assessed temperature and precipitation measurements for each hydrological year (July–June) relative to daily means over the period July 2000 to June 2015 (Figure 2). The climate exhibits strong hydrological seasonality, with well‐defined wet (Oct–Apr) and dry (May–Sept) seasons (Figure 2e). Hydrological year precipitation totals vary by ±15.3% of the annual mean (702 mm over 15 years), with notable wet years in 2001–2002 and 2002–2003 (both 15% above the mean) and dry years in 2003–2004 and 2007–2008 (−12% and −14% of the mean, respectively) (Figure 2d). Seasonal‐scale precipitation anomalies show that most hydrological years feature both positive and negative departures from the mean (Figure 2f). Anomalously wet conditions preceded the first site visits in March and August 2003 and again in March 2013, while precipitation deficits preceded the July 2004 visit, when we documented the *T. marmoratus* mortality event. Precipitation deficits also occurred during the March 2005, March 2008, March 2012, and March 2015 site visits (Figure 2f). An overall precipitation trend of −3.14 mm/year was experienced over the 15‐year period. The 15‐year trend in daily maximum temperature averages +0.07°C/year (Figure 2c), which is roughly equivalent to an isothermal elevation increase of 7–10 m/year across the Vilcanota region, and is consistent with multimodel projections of anthropogenically driven warming of the regional climate (IPCC, 2013). Modest interannual and seasonal variability in thermal maxima, ranging within ±1.5°C of mean conditions, was observed over the 15‐year period.

Observations suggest water tables may be lowering at our sites in the deglaciated zones. Direct surface‐level monitoring is not conducted at our study ponds, so for inference on water level variability and trends we use time series of precipitation observations, visual observations during site visits, and in‐pond temperature variations recorded by a datalogger. The diurnal temperature range of a datalogger deployed on 14 March 2008 at 30 cm below the surface in D5 Pond provides proxy indications of exposure from declining water levels and subsequent resubmersion cycles during seasonal transitions. In 2008, diurnal variations increased abruptly on 25 July and diminished on 24 November, indicating emergence and submergence, respectively (Figs S1 and 2f). In 2009, emergence occurred earlier, on 10 July, and at the time of retrieval on 2 August, the datalogger was 6 cm above the waterline. These observations are consistent with photodocumentation of water levels during late wet season (Mar–Apr) and dry season (Jul–Aug) site visits (not shown) and are consistent with the precipitation deficits occurring during these two site visits (Figure 2f).

### 3.2 Anuran population trends

**Figure 4:**
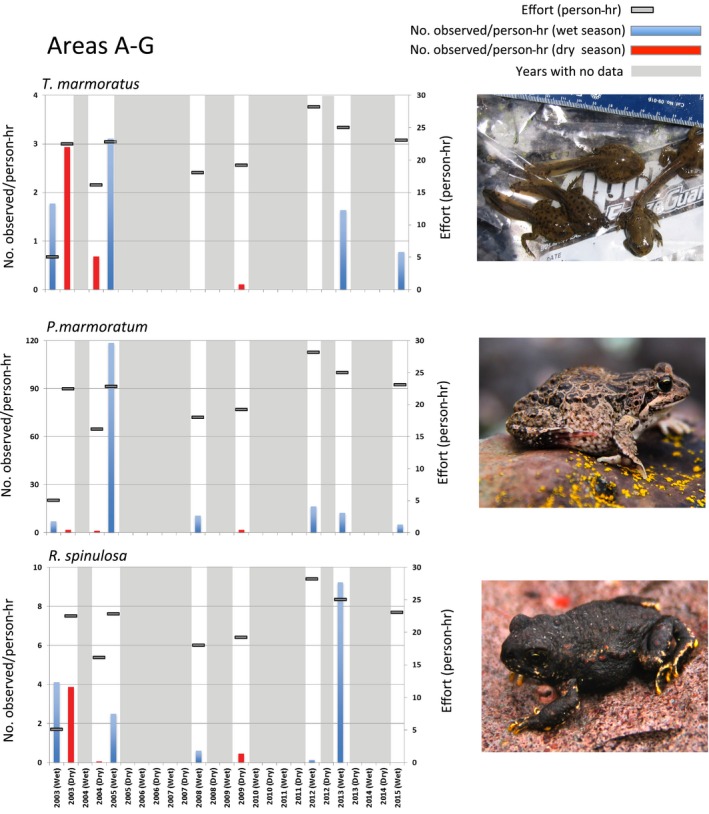
Comparison of *Telmatobius marmoratus*,* Rhinella spinulosa*, and *Pleurodema marmoratum* adult, juvenile, and tadpole (combined) populations recorded during the field expeditions between 2003 and 2015. Representative images of each species are shown. The relative effort (survey person‐hours) for areas A–G is shown as horizontal grey bars. Grey shaded areas denote years where no surveys were performed

High variability in anuran encounters was recorded during the site visits between 2003 and 2015, with all areas testing positive for *Bd*. Data are presented as adjusted encounters for each species: number of encountered individuals (tadpoles, juveniles, and/or adults combined)/effort (person‐hours; Figure 4). Because the vast majority of encounters were tadpoles, the numbers of metamorphic and postmetamorphic individuals were combined; however, the data for number of postmetamorphic individuals encountered are also provided in Tables S2 –S4. In 2003 and 2005, all three species were encountered; in general, dry season‐adjusted encounters are expected to be somewhat lower for anurans such as *P. marmoratum* and *R. spinulosa*, which breed in seasonal ponds (Figure 4). In July 2004, we observed fewer individuals of all species, largely due to fewer detections of tadpoles during the dry season (for *P. marmoratum* and *R. spinulosa*) and a mortality event at Area D (*T. marmoratus*) (Table S2). Between March 2008 and March 2012, three surveys were conducted; *P. marmoratum*‐adjusted encounters were frequent during the wet season and low in number during the dry season visits; *R. spinulosa* encounters were low in both the wet and dry seasons during this time. It should also be noted, however, as shown in Fig. S2, that we observed high variability in adjusted encounters when each area (A–G) was examined individually, indicating that each site has variations out of phase with the aggregated data for areas A–G. For example, *R. spinulosa* encounters varied across time and location: The majority of encounters occurred at Area C in 2003 (wet season), in Area A in 2003 (dry season), in Area B in 2005 (wet season), in Area C in 2008–2012 (wet and dry seasons), and in Area A in 2013 (Fig. S2). We did not encounter significant *R. spinulosa* or *P. marmoratum* mortality events during these 2 years.

We observed a major reduction in *T. marmoratus* encounters from 2008 to 2012 (Figures 4 and S2). *Telmatobius marmoratus* encounters initially decreased from 2003 to 2004 and increased in 2005 and then were far lower in 2008, 2009, and 2012 than in the previous years. Only two *T. marmoratus* were encountered during 55.8 hr of effort during the three survey years combined (Figure 4). This reduction, 2008–2012, was observed at all sites where *Telmatobius* had been previously found (Fig. S2). However, in March 2013, *T. marmoratus* encounters increased again.

The relationship of encounters or adjusted encounters to the amount of survey effort—comparing wet versus dry season—is presented as scatter plots in Fig. S3. There are relatively low correlations between encounters (*r* = .33 for *P. marmoratum*;* r* = .30 for *T. marmoratus*;* r* = .30 for *R. spinulosa*) or adjusted encounters (*r* = .26, −.04, .003*,* respectively) with the amount of survey effort.

In the highest areas, all three species have been documented at Area D (5,244 m), whereas only *P. marmoratum* has been documented at areas E (5,300–5,400 m) and F (5,348 m). At Area D, *T. marmoratus* relative abundance was 6 person‐hr^−1^ in 2003 (Figure 2). Encounters dropped considerably to 0.7 person‐hr^−1^ in 2004 following a mortality event, with 24 dead adult *T. marmoratus* recorded (no tadpoles; Table S2) (Seimon et al., 2007); dead individuals were not counted when measuring anuran abundance. This site visit was also preceded by a period of below‐average precipitation. Encounters of *T. marmoratus* then rebounded in 2005, also during a period of below‐average precipitation, although only tadpoles were encountered (no adults). During Area D surveys in 2008, 2009, and 2012, no *T. marmoratus* were encountered despite similar or higher levels of person effort than in previous years. All three of these survey years were also preceded by below‐average precipitation and a water table drop occurring in the dry seasons in 2008 and 2009 (Figure 2d,f). However, in 2013 and 2015, *T. marmoratus* once again were encountered, indicating abundance had increased (Figure 2g), and while the 2013 survey occurred during above‐average precipitation, the 2015 survey occurred during below‐average precipitation (Figure 2f).

While *P. marmoratum* was encountered at Area D during all surveys conducted between 2003 and 2015 (Figure 2h), *R. spinulosa* was encountered only in 2005, indicating that its range expansion to Area D was likely temporary (Figure 2i). In Area E, *P. marmoratum* relative abundance was stable during wet seasons and greatly reduced (fewer tadpole encounters) during dry seasons, as expected between 2003 and 2015 (Table S3). However, in Area F, *P. marmoratum* was not encountered in 2008 and 2009, which is also when we observed die‐off of cushion plants and seasonal desiccation of the pond (Figures 3 and S2). By 2015, wet season *P. marmoratum* abundance in Area F had rebounded and was similar to the encounters observed in Area E, although the cushion plants did not recover (Fig. S2).

**Figure 5:**
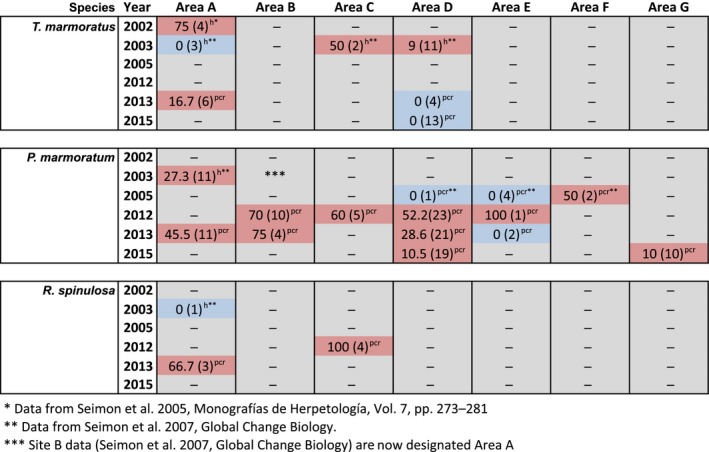
Summary of *Batrachochytrium dendrobatidis* (*Bd*) results for areas A–G from 2002 to 2015 for *Telmatobius marmoratus*,* Pleurodema marmoratum*, and *Rhinella spinulosa*. Shown are percent of samples that tested positive for *Bd* by either histology (^h^) or by PCR (^pcr^), and number of individuals tested. Pink boxes designate which years from the corresponding area (A–G) were positive for *Bd*; blue corresponds to areas that tested negative for *Bd*. Dash lines in grey boxes indicate where no samples were collected for *Bd* testing
% *Bd*‐positive (number tested); *Data from Seimon et al. (2005); **Data from Seimon et al. (2007); ***Site B data (Seimon et al., 2007) is now designated Area A.

Despite the continued presence of *Bd*,* T. marmoratus* encounters increased in 2013 and 2015. In surveys from 2012 to 2015, we assessed *Bd* prevalence in anurans from areas A–G and compared these results to previous data collected between 2002 and 2005 (Seimon et al., 2005, 2007). We collected 139 swab samples in total: 136 samples from apparently healthy anurans and three from dead individuals encountered during surveys. Toe clips from all three anuran carcasses were positive for *Bd* by PCR. The dead anurans were a juvenile *P. marmoratum* with 2,246 (444 STDEV) copies of the ITS1‐5.8S region per sample, a juvenile *R. spinulosa* with 231,330 (20,052 STDEV) copies per sample—both collected under the same rock in Area C in 2012—and a dead adult *P. marmoratum* with 1,713,901 (760,439 STDEV) copies per sample, collected in Area D in 2013. The ITS1‐5.8S region of the *Bd* genome is multicopy, and copy number varies considerably between strains. If we assume an average of 77 copies per zoospore (Seimon, Ayebare, et al., 2015), the zoospore range would be equivalent to 29–22,258 zoospores/sample. However, at the time of collection, the carcasses were desiccated, which may have impacted the quality of the DNA because of degradation, leading to an underestimation of copy number. Of the 136 swab samples collected from live individuals, 106 were from adult and juvenile *P. marmoratum*; seven were from adult and juvenile *R. spinulosa*; and 23 were from *T. marmoratus* tadpoles (no adults or juveniles were encountered in 2013 and 2015). The percent positive for *Bd* for each species and number of individuals tested in each area (A–G) are presented in Figures 5 and 2j,k. In Area D, both *T. marmoratus* (in 2003) and *P. marmoratum* (in 2012, 2013, and 2015) have tested positive for *Bd,* indicating that this pathogen has been in this environment and series of connected ponds for at least 12 years (Figure 2j,k). *Bd* and the disease chytridiomycosis were detected in *T. marmoratus* in Area D in 2003, 1 year prior to the mortality event in 2004, and were detected in *P. marmoratum* while *T. marmoratus* encounters had increased in 2013 and 2015 (Figure 2). Overall, individuals from all seven areas (A–G) and all three species tested positive for *Bd*. Individuals in areas A and C have repeatedly tested positive over 11 and 9 years, respectively. Individuals tested positive from Area B in 2012 and 2013, Area F in 2005, and Area G in 2015. In Area E in 2012, we detected *Bd* from an energetic and healthy‐appearing adult *P. marmoratum* encountered in deglaciated terrain at 5,314 m.

## 4 Discussion

This study provides insights into the temporal effects of, and relationships between, global change phenomena as experienced by amphibians monitored for more than a decade in the tropical high Andes. These include the following: climate warming driving deglaciation, expansion of amphibians into new ecological niches, dynamic changes in amphibian habitat, spread of the pathogenic *Bd* fungus, and how amphibian populations are responding. The early findings from our high Andean anuran monitoring, reported in Seimon et al. (2007), suggested populations in the Vilcanota may be facing imminent collapse; however, subsequent monitoring has shown that this is not the case, and trends indicative of decline may be reversing.

Our results provide baseline data on the variability in population fluctuations of three anuran species in rapidly changing high‐alpine habitats where *Bd* has been present for over a decade. Amphibian species in the genus *Telmatobius* are among the most threatened in Peru and the Andes in general, and chytridiomycosis has been cited as a key threat to these vulnerable populations (Jarvis et al., 2015; von May et al., 2008). Long‐term monitoring is needed to better understand and assess the threats contributing to the ongoing decline in these and other amphibian species (Catenazzi & von May, 2014). The paucity of site visits, the absence of data on natural population fluctuations in the landscape prior to the introduction of *Bd*, and difficulties in conducting systematic data collection at all sites over the 12‐year observational period make statistical testing and multivariate analysis among the disparate datasets problematic. Nevertheless, important inferences can be derived from our data and are discussed below.

### 4.1 Anuran habitat changes

In the Cordillera Vilcanota, deglaciation is continually creating new amphibian habitat where rapid ecological successions follow ice retreat. In Area E, two new ponds (E 2005 Pond and E 2008 Pond) emerged from retreating ice—at 5,369 m in 2005 and at 5,383 m in 2008—and are examples of several that have formed over the past decade. No anurans have been observed at these ponds to date, but *P. marmoratum* has colonized E7 Pond located 20 meters from E 2008 Pond. Continued monitoring will reveal whether range expansion proceeds as habitat and food resources develop.

Ecological successions and water table drops from glacial retreat are also changing anuran habitat. Recently developed ponds and habitat colonized by anurans at the highest areas, D–F, continue to undergo successions; this includes progressive die‐offs of cushion plants. Our observations indicate that this is tied to the seasonal drop in water table occurring each dry season that may be increasing in amplitude. Negative precipitation anomalies from 2004 to 2009 may partially explain the initial water table decline, although subsequent precipitation increases have failed to reinvigorate desiccating wetland habitat and the water table drop continues to expose the deeper layers and soil root matrix of the underlying peat to an oxygenated and drier environment. This may be hindering growth and causing plant mortality of the vegetation, as has been observed at other sites in the Andes (Cooper, Kaczynski, Slayback, & Yager, 2015; Yager, 2015).

The Area D ponds emerged from the ice margin between 1880 and 1931 (Seimon et al., 2007), and the glacier continues receding at a mean rate of 13.1 m/year. Andean peatlands, such as the *D. muscoides*‐dominated peatland in the Sibinacocha watershed, are azonal vegetation systems that depend on hydrological support year round, which may come from ground water, precipitation, or glacial outflow (Squeo, Warner, Aravena, & Espinoza, 2006). Melting glaciers provide a persistent inflow of water necessary for the maintenance and sustained growth of *D. muscoides‐*dominated peatland vegetation (Benavides, Vitt, & Wieder, 2013), and periglacial lakes in the Cordillera Vilcanota region are widely observed to experience water level declines following the initial melt‐water pulse as retreating ice diminishes glacial provision of inflow (Hanshaw & Bookhagen, 2013). If the dominant source of water for a peatland system shifts or disappears—such as may occur with glacial runoff and outflow—then the peatland system may seasonally desiccate and eventually die‐off if no other hydrological support continues. As cushion plants age and die, it is common for tussock grasses to colonize their centers in general alpine cyclical succession (Mark & Bastow, 2005) as observed in this study. The complete loss of glaciers driven by climatic changes is expected to have an overall effect of reducing *Distichia*‐dominated habitat and wetlands (Anthelme, Cavieres, & Dangles, 2014; Benavides et al., 2013; Cooper et al., 2015; Herrera & Anthelme, 2015), for which these high‐elevation anuran species are dependent for breeding and survival.

### 4.2 Anuran survival in context of *Bd* prevalence and environmental changes

The observations reported here may be informative to conservation planning. *P. marmoratum* and *R. spinulosa* are listed as Least Concern species by the IUCN Red List of Threatened Species; *P. marmoratum* populations are thought to be decreasing, while *R. spinulosa* population trends are unknown, and both are known to have broad ranges (IUCN, 2015a 2015 and IUCN, 2015b 2015). The present study shows that in the Cordillera Vilcanota, amphibian populations are surviving despite the sustained and widespread presence of *Bd* infection in all species for over a decade and confirmed occurrence of chytridiomycosis in *T. marmoratus* and *P. marmoratum* (Seimon et al., 2005, 2007). The results of this study may therefore have implications for the risk assessments of these three species, at least in the upper levels of their elevational distribution.

Repeated observations and both wet and dry season visitation revealed different patterns of anuran abundance among the three species. Visual encounter surveys indicate that *R. spinulosa* encounters are highly variable in time and location. *R. spinulosa* was only found once in the deglaciated zone, in 2005 (Seimon et al., 2007), and its temporary expansion into the deglaciated area may reflect the variability in encounters we documented at other sites. *P. marmoratum* encounters were relatively stable in the deglaciated zone between 2003 and 2015, except at the highest site (E8 Pond at 5400 m) where it was found only in 2005. However, the repeated observations of *P. marmoratum* at other Area E ponds in the deglaciated zone below E8 since 2005 also indicate that the multidecadal upward range extension of this species continues. These results, paired with the observations of *R. spinulosa*, indicate that expansion of each species to the absolute highest recorded sites is temporary and parallels the highly dynamic metapopulation fluctuations often encountered in these amphibians (see e.g., Halloy & Laurent, 1988).

### 4.3 Rebound in *Telmatobius marmoratus*

*Telmatobius marmoratus* has the broadest distribution of any species in this genus and is classified as Vulnerable based on the projection of future decline (criterion A3) due to threats imposed by overharvesting for food consumption, habitat loss, water pollution, and chytridiomycosis (IUCN, 2015c 2015). After almost no *T. marmoratus* were encountered from 2008 to 2012, the species was again observed in 2013 and 2015, indicating that despite the presence of *Bd* in the environment for over a decade, the observed reduction did not portend local extinction. Among implications of these observations is the possibility that environmental heterogeneity, genetic diversity, or resistance to chytridiomycosis through changes in their skin microbiome may provide mechanisms for small pockets of amphibian populations to survive—and then repopulate—potentially with more resistant genes. The high population variability across a heterogeneous landscape may provide opportunities for repopulation to occur postdecline. For example, clustered water bodies, streams and other watercourses, shady rocks, and vegetation corridors can act as refuges, sources, breeding grounds, or stepping‐stones in migrations of amphibians from one area to another. Such conditions would imply high genetic diversity in resident amphibians because of mixing of populations, which may allow “escape” or adaptation for certain diseases or extreme conditions. Studies in Patagonia have shown the plasticity of *Atelognathus patagonicus*, a relative of *Telmatobius*, which is able to change from terrestrial to aquatic forms, and vice versa, depending on environmental conditions (Cuello, Úbeda, Bello, & Perotti, 2014). Future investigations into genetic variability, environmental heterogeneity, and determining whether changes in amphibian skin microbiome have been occurring (Harris et al., 2009; Jani & Briggs, 2014) will be essential for assessing how well these anuran species will withstand and survive continued climate‐driven and anthropogenic landscape changes. This may lend support for hypothesized advantages of landscape heterogeneity, population dynamics (Anderson, Clark, & Sheldon, 2012; Poiani, Richter, Anderson, & Richter, 2000), and genetic variability allowing evolution and adaptation to changing conditions (Matthews & Boltz, 2012; Reed, Schindler, & Waples, 2010).

### 4.4 The role of *Bd* in *Telmatobius* mortalities in the Peruvian Andes

Data on natural fluctuations of anuran populations prior to the discovery of *Bd* in our field region in 2002 do not exist; therefore, we cannot determine whether the multiyear reduction in *T. marmoratus* is consistent with environmental fluctuations associated with below‐average precipitation and habitat change from dynamically changing landscape, rather than from an invasive pathogen, or the combination of these variables. Predation by trout and endemic carnivores such as Culpeo fox (*Lycalopex culpaeus*), pampas cat (*Leopardus pajeros*), and avifauna such as mountain caracara (*Phalcoboenus megalopterus*) seems unlikely to be drivers of the *Telmatobius* population reduction as they have coexisted with amphibians in the region for decades (for introduced trout) to millennia, or longer. Human consumption of *T. marmoratus* was reported at Area A, but not at other sites. There is no evidence for the use of agrochemicals at our study sites, which all lie above cultivated terrain. Recently, a high prevalence of co‐infection of *Bd* with ranavirus has been reported in *T. marmoratus* found in the San Pedro Market in Cusco, as well as other species at a nearby field site in Kosñipata Valley near Manu National Park (Warne et al., 2016). However, we have no evidence pointing to the presence of ranavirus in our study site. Liver tissue from encountered carcasses (five *P. marmoratum* and one *T. marmoratus*) collected in 2014 from areas A, B, D, and E were tested for ranavirus, and all samples were negative (K. Reider and T. Seimon, unpublished results). However, *Bd* has been found at all areas surveyed (A–G), of which three (A, C, D) remained positive when sampled over more than a decade (2003–2015). Chytridiomycosis has been documented in *T. marmoratus* from areas A, C, and D, and *Bd* infection has also been found at Area B. Areas A–D are also where reductions of this species have been documented.

*Batrachochytrium dendrobatidis* has been proposed to have spread across the Andes in three epidemic waves in the late 1970s and early 1980s (Lips, Diffendorfer, Mendelson, & Sears, 2008). The timeline and rate of spread support the hypothesis that *Bd* propagated from the northern Peruvian Andes around 1999, where the earliest records of declines associated with *Bd* occur, to southern Peru (2002) where the most recent anuran declines have been occurring (Catenazzi & von May, 2014; Catenazzi et al., 2011; Lips et al., 2008; Seimon et al., 2005, 2007; Venegas, Catenazzi, Siu‐Ting, & Carrillo, 2008). Studies conducted 80 km NW of the Cordillera Vilcanota, in upper Manu National Park (1,200–3,700 m), identified a 36% decrease in anuran species between 1999 and 2009, including two species of *Telmatobius* (Catenazzi et al., 2011). *Telmatobius timens*, commonly encountered until 1999, was not observed in surveys from 2007 to 2009, and local herders reported massive die‐offs of *Telmatobius* frogs in 2004 (Catenazzi & von May, 2014). Climatic factors did not appear to be drivers of amphibian declines in the Manu highlands (Catenazzi et al., 2014). The increased prevalence of *Bd* over time correlated with the increased percentage of species that disappeared, suggesting that *Bd* was likely the driving factor of the amphibian declines in the southern Peruvian Andes between 1999 and 2009 (Catenazzi et al., 2014). These data are consistent with local herders' reports in the Cordillera Vilcanota of the disappearance of frogs in 2002; *Telmatobius* mortalities in 2004; and detection of *Bd* and the disease chytridiomycosis*,* and amphibian declines during the same decade (Seimon et al., 2005, 2007). It is noteworthy that documentation of chytridiomycosis and *Telmatobius* mortalities occurred during the dry seasons (Seimon et al., 2005, 2007), and the reduction in encounters of *Telmatobius* occurred in years with reduced precipitation. Dry season conditions in the high Andes can increase *Bd* prevalence in populations, most likely due to reduced flow of water into ponds (Catenazzi et al., 2013): Lowering the water table can result in higher thermal amplitudes, anoxic conditions and concentration of organic matter, zoospores, and anurans, all of which could augment growth conditions for *Bd*, cause stress in amphibians, and make outbreaks of chytridiomycosis or other pathogens and parasites more likely (Catenazzi et al., 2013). *Telmatobius* tadpoles can also act as disease reservoirs for other life stages making *Telmatobius* vulnerable to chytridiomycosis (Catenazzi et al., 2013).

Ongoing studies monitoring *Bd* throughout the wet and dry seasons are underway and could identify whether pathogen loads and prevalence are higher as the water table drops and water flow is reduced, and if mortality events correlate with higher *Bd* prevalence. Critical to understanding the long‐term conservation threat of *Bd* to *Telmatobius* and other threatened genera in South America is determining whether *T. marmoratus* and sympatric species such as *P. marmoratum* and *R. spinulosa* have the potential to develop, or have already developed, innate tolerance to *Bd* infection. This has been observed for several anuran species in Africa (Seimon, Ayebare, et al., 2015).

In summary, deglaciation, anuran habitat, and landscape changes all continue to progress at a very rapid rate in the Cordillera Vilcanota. Three monitored anuran species remain extant despite the sustained occurrence of *Bd* infections for over a decade. In the wake of glacial recession, cushion peatlands are continually forming and transforming by ecological successions, presenting resident anurans with rapidly changing habitats. Developing water bodies in the wake of glacial recession evolve into suitable habitat for anurans, which over time expand their range and colonize. However, as the glaciers continue to recede, some perennial ponds lose stable inflow of water from glacial melt. Desiccating perennial ponds promote successions from wetland to dryland vegetation, can promote oxygen depletion in the aquatic habitat, and, as they transition into seasonally dry ponds, may not persist long enough for complete tadpole development. Additional research is needed to understand more about the plasticity and genetic variability of these anuran species; the contributions of inflow from precipitation versus glacial melt in sustaining high Andean amphibian habitats; the degree of infiltration and amplification of the seasonal water table drop over time during the dry and wet seasons; whether the drop in water table correlates with increased *Bd* prevalence and infection; and how perennial to seasonal pond transitions may affect food resources, thermal regime, chemistry, oxygen content, and tadpole development. Such information will lead to a better understanding of the long‐term viability of these ponds as suitable amphibian habitat and the consequences on biodiversity once these glaciers are gone (Quenta et al., 2016) and may help inform conservation strategies such as cushion bog peatland cultivation and expansion. Ancient traditional methods of *bofedale* expansion (Squeo et al., 2006; Yager, 2009) could be utilized to help restore and expand anuran habitat and food resources, and provide connectivity of wetlands as a measure to preserve these species. Protecting the connectivity of wetlands in the Cordillera Vilcanota and other high‐alpine regions will be essential to ensure these anurans and other species can adapt and prevail as climate change continues to reshape this environment.

## Conflict of Interest

None declared.
