# Supplementary figures and images for: Long‐term monitoring of tropical alpine habitat change, Andean anurans, and chytrid fungus in the Cordillera Vilcanota, Peru: Results from a decade of study
Source: Ecol Evol. 2017 Feb 7;7(5):1527–40. doi: 10.1002/ece3.2779 (PMC5330894; doi:10.1002/ece3.2779)

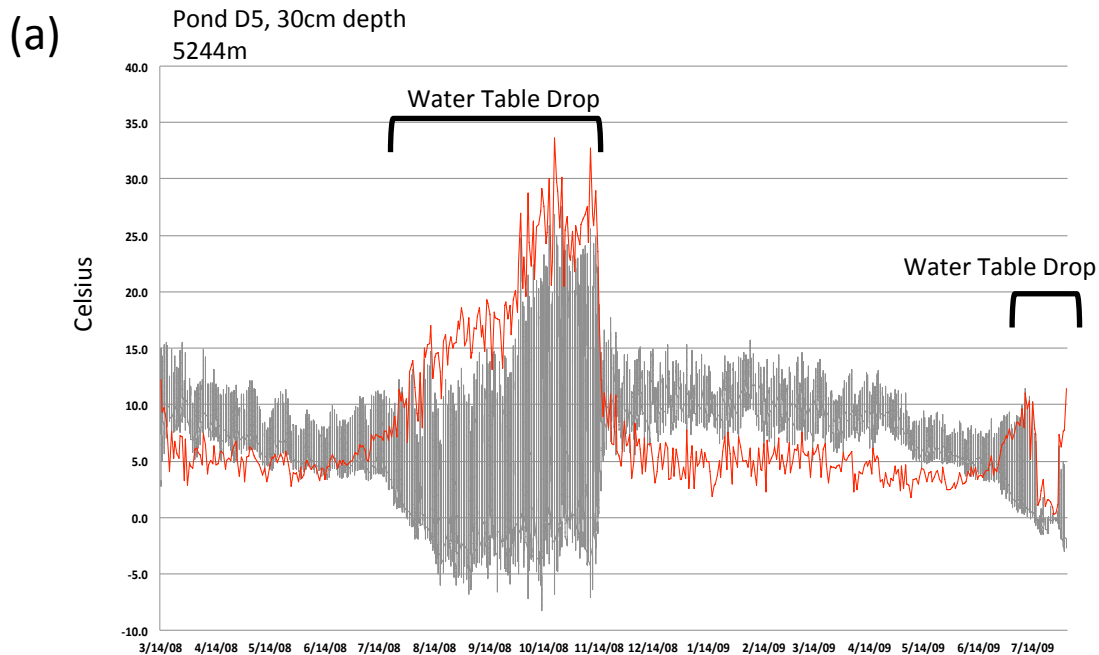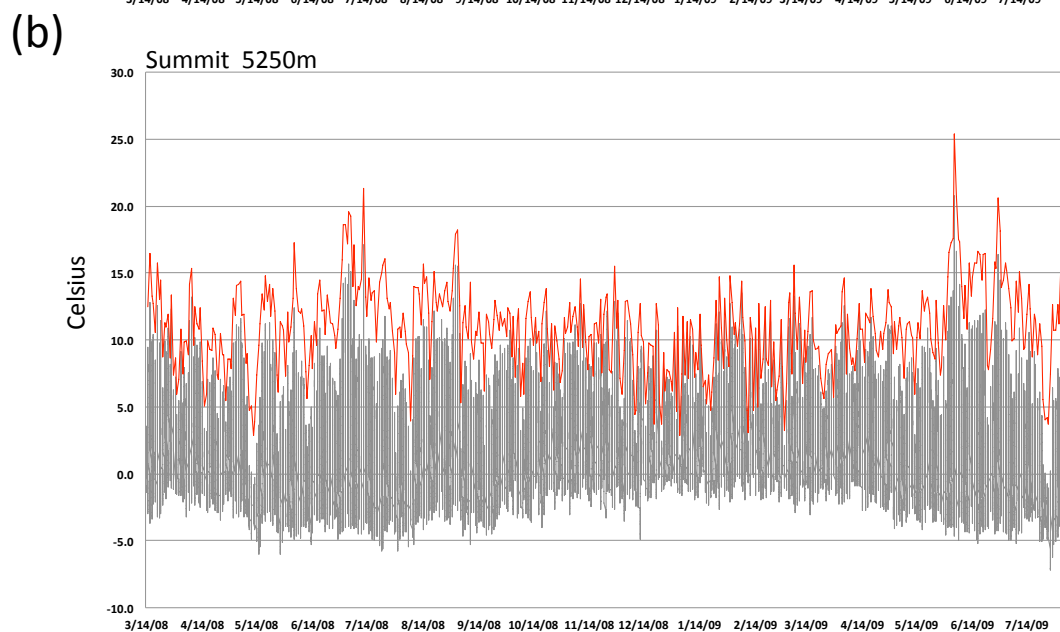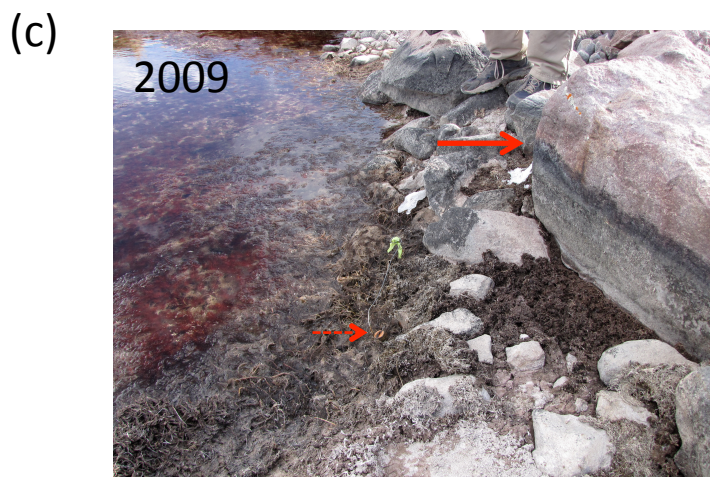

Fig S1

Supplement: Supplementary file 1 [file ECE3-7-1527-s001.pdf]

(a)

*P. marmoratum*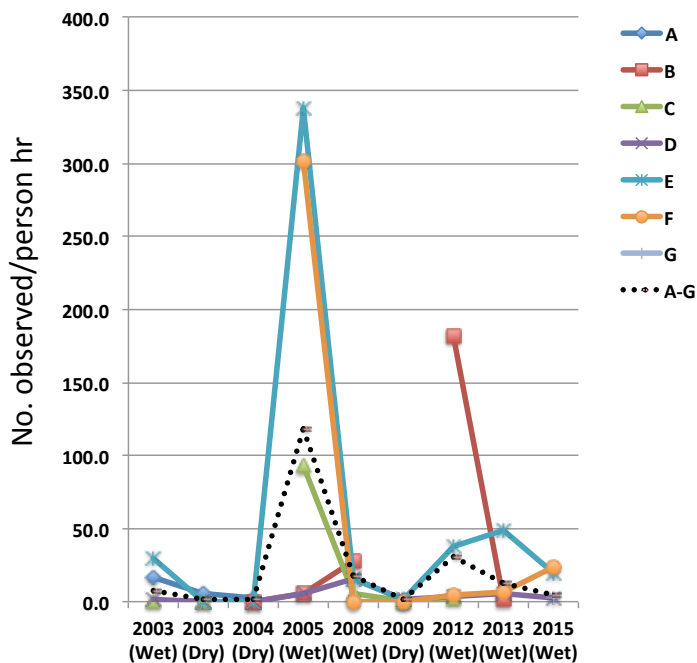

(b)

*T. marmoratus*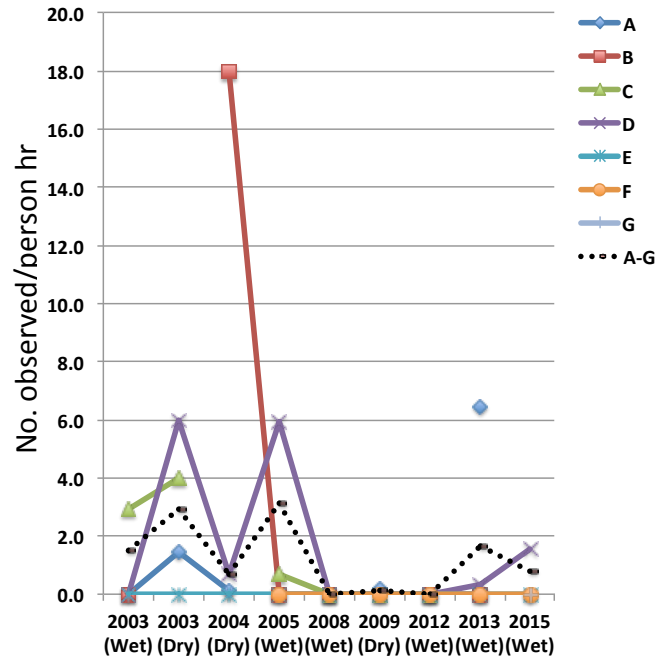

(c)

*R. spinulosa*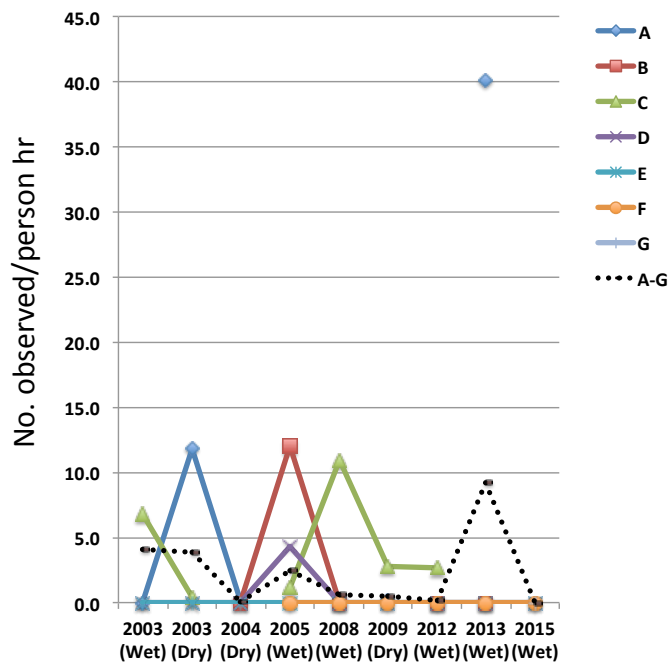

(d)

Amount of effort

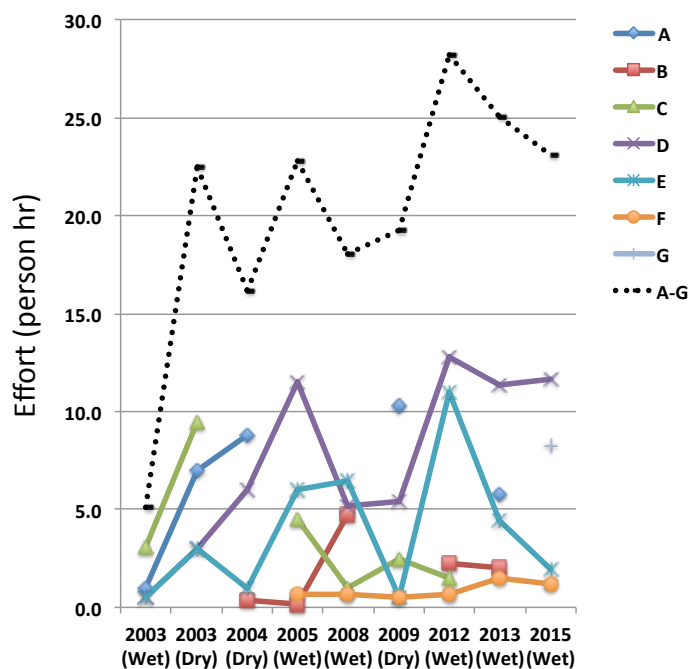

Supplement: Supplementary file 2 [file ECE3-7-1527-s002.pdf]

● Wet season  
● Dry season

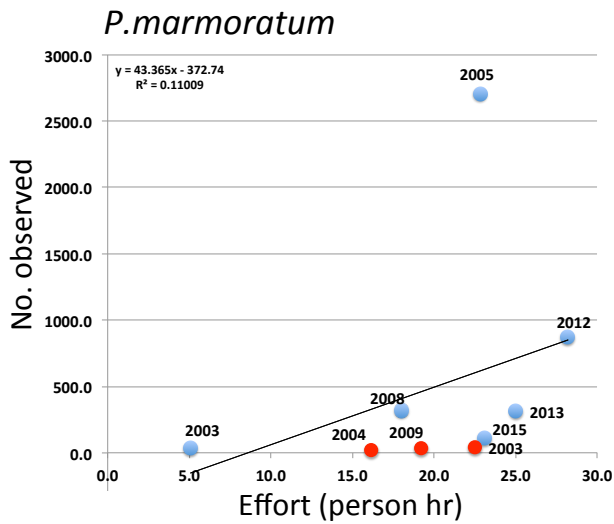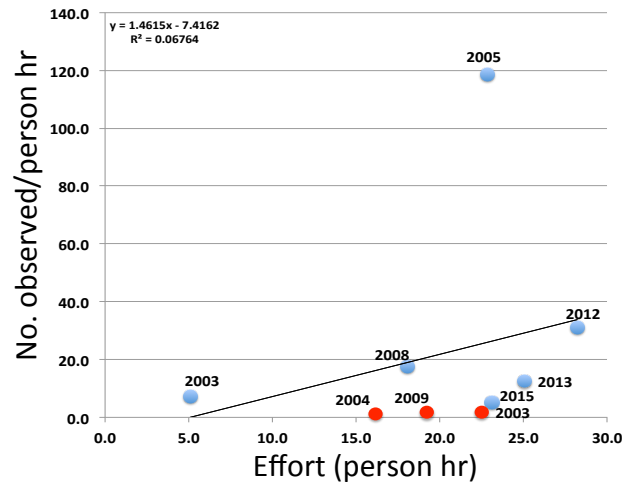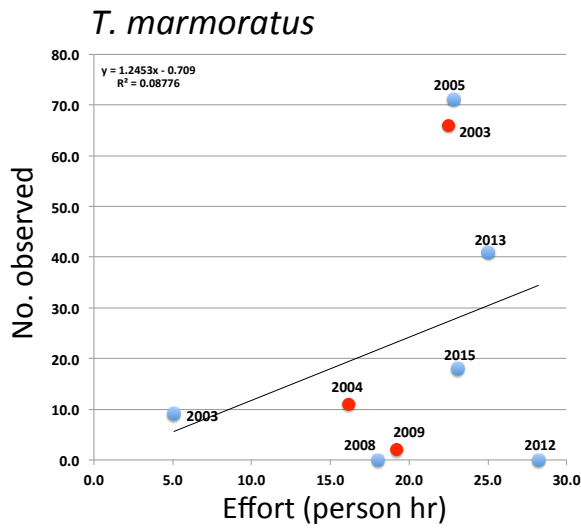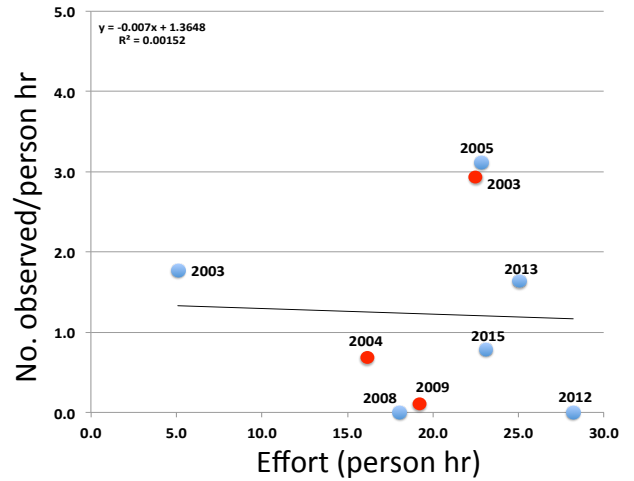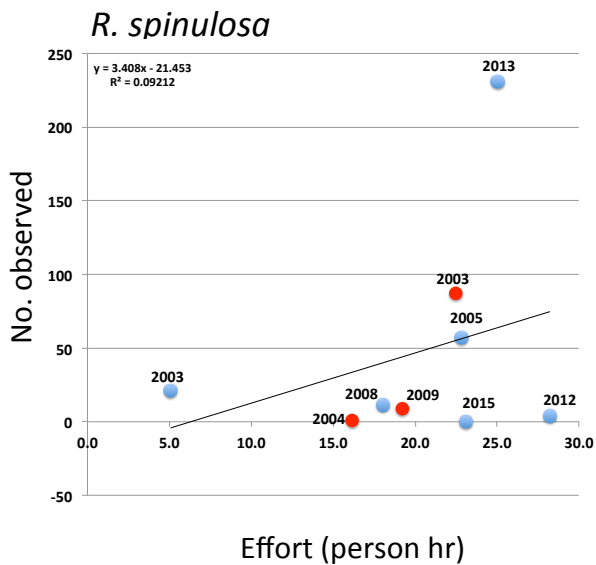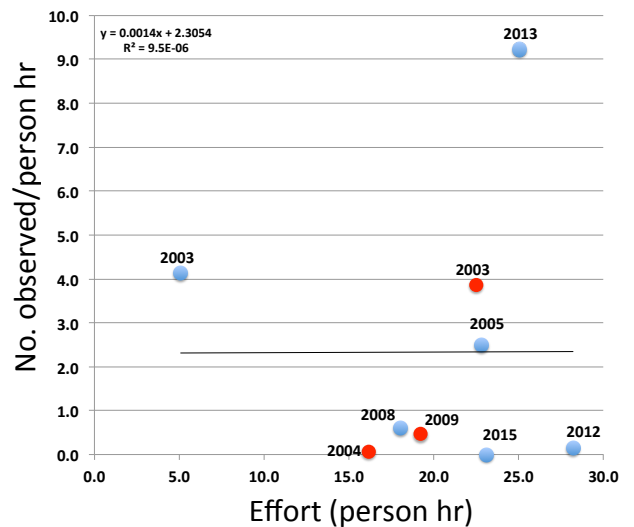

Fig S3

Supplement: Supplementary file 3 [file ECE3-7-1527-s003.pdf]
