# Supplementary material for: Long‐term monitoring of tropical alpine habitat change, Andean anurans, and chytrid fungus in the Cordillera Vilcanota, Peru: Results from a decade of study
Source: Ecol Evol. 2017 Feb 7;7(5):1527–40. doi: 10.1002/ece3.2779 (PMC5330894; doi:10.1002/ece3.2779)
Supplement: Supplementary file 4 [file ECE3-7-1527-s004.docx]

**Supplementary Table S1. Locations of visual encounter surveys at Areas A-G.**

| **Survey area** | **Description** | **Elevation (m)** | **PSAD 56 UTM Easting** | **PSAD 56 UTM Northing** | **Latitude** | **Longitude** |
| --- | --- | --- | --- | --- | --- | --- |
| Area A, Rio Pitumarca catchment | Ausangate Valley | 4605 | 0257431 | 8469654 | -13.8359 | -71.2460 |
|  | Ausangate Valley | 4650 | 0258140 | 8470436 | -13.8289 | -71.2393 |
|  | Chillca | 4409 | 0258277 | 8464458 | -13.8829 | -71.2386 |
|  | Chillca | 4332 | 0258139 | 8464636 | -13.8813 | -71.2395 |
|  | Chillca | 4362 | 0261503 | 8465358 | -13.8750 | -71.2087 |
|  | Hda.Uyuni | 4410 | 0262805 | 8467116 | -13.8593 | -71.1965 |
|  | Hda. Uyuni, A2, | 4400 | 0262484 | 8466608 | -13.8638 | -71.1995 |
|  | Hda. Uyuni, A2 (spring), | 4422 | 0262860 | 8467060 | -13.8598 | -71.1960 |
|  | Condor Pass west side | 5027 | 0270202 | 8469405 | -13.8392 | -71.1279 |
| Area B, Murmurani catchment | Pumachunta transect (5X300m) start | 4975 | 0276637 | 8469977 | -13.8352 | -71.0681 |
|  | Condor Pass | 5181 | 0271057 | 8469895 | -13.8348 | -71.1199 |
|  | Pumachunta transect (5X300m) end | 4957 | 0276735 | 8469697 | -13.8376 | -71.0672 |
|  | Local Farm | 4983 | 0274322 | 8472449 | -13.8120 | -71.0895 |
|  | Murmarani | 4875 | 0275676 | 8471916 | -13.8169 | -71.0771 |
|  | Murmarani | 4921 | 0276084 | 8469684 | -13.8371 | -71.0735 |
|  | Alto Murmurani | 5144 | 0271964 | 8471954 | -13.8163 | -71.1114 |
|  | Alto Murmurani | 5009 | 0274277 | 8471922 | -13.8168 | -71.0900 |
| Area C, Jatunriti catchment | Lga Sibinacocha | 4872 | 0277000 | 8473000 | -13.8072 | -71.0643 |
|  | Pampa N of Lga Sibinacocha | 4929 | 0276107 | 8475132 | -13.7879 | -71.0728 |
|  | Pampa N of Lga Sibinacocha | 4872 | 0276075 | 8474738 | -13.7915 | -71.0731 |
|  | Between basecamp and high camp | 5187 | 0275772 | 8477645 | -13.7652 | -71.0757 |
|  | Near basecamp | 5000 | 0275637 | 8476396 | -13.7765 | -71.0771 |
|  | South of basecamp | 4945 | 0275661 | 8475370 | -13.7857 | -71.0769 |
|  | South of basecamp | 4914 | 0275805 | 8474719 | -13.7916 | -71.0756 |
|  | South of basecamp | 4909 | 0275669 | 8474191 | -13.7964 | -71.0769 |
|  | South of basecamp | 4950 | 0276085 | 8473294 | -13.8045 | -71.0732 |
|  | South of basecamp | 4923 | 0275855 | 8473790 | -13.8000 | -71.0753 |
|  | South of basecamp | 4897 | 0275945 | 8474030 | -13.7979 | -71.0744 |
|  | South of basecamp | 4901 | 0275965 | 8474058 | -13.7976 | -71.0742 |
|  | South of basecamp | 4938 | 0275812 | 8474078 | -13.7974 | -71.0756 |
|  | South of basecamp | 4941 | 0275785 | 8474166 | -13.7966 | -71.0759 |
|  | Near basecamp | 4943 | 0275675 | 8474922 | -13.7898 | -71.0768 |
|  | Near basecamp | 4939 | 0275573 | 8475458 | -13.7849 | -71.0777 |
|  | Near basecamp | 4939 | 0275558 | 8475602 | -13.7836 | -71.0779 |
|  | Rincon Trail-S of Sibinacocha Basecamp | 4909 | 0275669 | 8474191 | -13.7964 | -71.0769 |
| Area D, Deglaciated permanent ponds | Ponds D1 - D6 | 5244 | 0275305 | 8477875 | -13.7631 | -71.0800 |
|  | Pond D7 - D8 | 5245 | 0274961 | 8477366 | -13.7640 | -71.0815 |
| Area E, Deglaciated south side of pass | Between moraine ridges in pass area | 5302 | 0275039 | 8478137 | -13.7607 | -71.0825 |
|  | E0 pond | 5294 | 0274897 | 8478000 | -13.7619 | -71.0838 |
|  | E1 pond | 5360 | 0274716 | 8478312 | -13.7557 | -71.0835 |
|  | E2 pond | 5369 | 0274902 | 8478686 | -13.7557 | -71.0837 |
|  | E3 pond | 5371 | 0274914 | 8478748 | -13.7551 | -71.0836 |
|  | E4 pond | 5371 | 0274903 | 8478746 | -13.7552 | -71.0837 |
|  | E5 pond | 5372 | 0274898 | 8478741 | -13.7552 | -71.0837 |
|  | E6 pond | 5376 | 0274875 | 8478833 | -13.7544 | -71.0839 |
|  | E7 pond | 5381 | 0274755 | 8478740 | -13.7552 | -71.0850 |
|  | E8 pond | 5400 | 0274614 | 8478847 | -13.7542 | -71.0863 |
|  | E9 pond | 5388 | 0274568 | 8478791 | -13.7547 | -71.0868 |
|  | E10 pond | 5323 | 0275102 | 8478362 | -13.7586 | -71.0819 |
|  | E11 pond | 5328 | 0275095 | 8478368 | -13.7586 | -71.0819 |
|  | E 1997 Pond | 5345 | 0274898 | 8478487 | -13.7574 | -71.0836 |
|  | Okarcocha transect (5X300m) start | 5314 | 0275002 | 8478065 | -13.7613 | -71.0828 |
|  | Okarcocha transect (5X300m) end | 5273 | 0275057 | 8477835 | -13.7634 | -71.0823 |
|  | Rititica transect (5X300m) start | 5282 | 0275189 | 8477539 | -13.7661 | -71.0811 |
|  | Rititica transect (5X300m) end | 5185 | 0275316 | 8477312 | -13.7682 | -71.0800 |
|  | E 2005 Pond | 5369 | 0274170 | 8478796 | -13.7547 | -71.0903 |
|  | E 2008 Pond | 5383 | 0274693 | 8478718 | -13.7554 | -71.0856 |
| Area F, Deglaciated north side of pass | Pond | 5348 | 0274463 | 8479214 | -13.7509 | -71.0878 |
| Area G | G1 | 4941 | 0282050 | 8475059 | -13.7888 | -71.0179 |
|  | G2 | 4955 | 0282110 | 8475014 | -13.7893 | -71.0175 |
|  | G3 | 4956 | 0282078 | 8474885 | -13.7902 | -71.0177 |
|  | G4 | 4955 | 0282078 | 8474814 | -13.7910 | -71.0176 |
|  | G5 | 4954 | 0281909 | 8474634 | -13.7927 | -71.0192 |
