# Supplementary material for: Long‐term monitoring of tropical alpine habitat change, Andean anurans, and chytrid fungus in the Cordillera Vilcanota, Peru: Results from a decade of study
Source: Ecol Evol. 2017 Feb 7;7(5):1527–40. doi: 10.1002/ece3.2779 (PMC5330894; doi:10.1002/ece3.2779)
Supplement: Supplementary file 5 [file ECE3-7-1527-s005.docx]

**Supplementary Table S2.** Results of visual encounter surveys for *T. marmoratus* at Areas A-G, and number of individuals tested and prevalence of *Bd* at each location.

* Indicates samples that were tested by histology for *Bd*, whereas those without the asterisk were tested by PCR.

|  | ***Telmatobius marmoratus*** | **3-7 March, 2003 (Wet)** | **8-15 August, 2003 (Dry)** | **17-31 July, 2004 (Dry)** | **16-20 March, 2005 (Wet)** | **10-20 March, 2008 (Wet)** | **27 July -6 August, 2009 (Dry)** | **2-13 April, 2012 (Wet)** | **19-26 March, 2013 (Wet)** | **15-25 March, 2015 (Wet)** |
| --- | --- | --- | --- | --- | --- | --- | --- | --- | --- | --- |
| **Area A** | No. obs/survey person hr | 0 | 1.4 | 0.1 | 0 | 0 | 0.2 | 0 | 6.4 | 0 |
|  | No. individuals | 0 | 10 | 1 | 0 | 0 | 2 | 0 | 37 | 0 |
|  | No. Post-metamorphosis | 0 | 0 | 0 | 0 | 0 | 0 | 0 | 0 | 0 |
|  | Survey person hrs | 1.0 | 7.0 | 8.8 | 0 | 0 | 10.3 | 0 | 5.8 | 0 |
|  | No. dead individuals | 0 | 0 | 0 | 0 | 0 | 1.0 | 0 | 0 | 0 |
|  |  |  |  |  |  |  |  |  |  |  |
|  | No. animals tested | 0 | 3.0* | 0 | 0 | 0 | 0 | 0 | 6.0 | 0 |
|  | % positive for *Bd* | - | 0 | - | - | - | - | - | 16.7 | - |
|  |  |  |  |  |  |  |  |  |  |  |
| **Area B** | No. obs/survey person hr | 0 | 0 | 18.2 | 0 | 0 | 0 | 0 | 0 | 0 |
|  | No. individuals | 0 | 0 | 6 | 0 | 0 | 0 | 0 | 0 | 0 |
|  | No. Post-metamorphosis | 0 | 0 | 0 | 0 | 0 | 0 | 0 | 0 | 0 |
|  | Survey person hrs | 0 | 0 | 0.33 | 0.17 | 4.7 | 0 | 2.3 | 2.0 | 0 |
|  | No. dead individuals | 0 | 0 | 0 | 0 | 0 | 0 | 0 | 0 | 0 |
|  |  |  |  |  |  |  |  |  |  |  |
|  | No. animals tested | 0 | 0 | 0 | 0 | 0 | 0 | 0 | 0 | 0 |
|  | % positive for *Bd* | - | - | - | - | - | - | - | - | - |
|  |  |  |  |  |  |  |  |  |  |  |
| **Area C** | No. obs/survey person hr | 2.9 | 4.0 | 0 | 0.7 | 0 | 0 | 0 | 0 | 0 |
|  | No. individuals | 9 | 38 | 0 | 3 | 0 | 0 | 0 | 0 | 0 |
|  | No. Post-metamorphosis | 2 | 9 | 0 | 0 | 0 | 0 | 0 | 0 | 0 |
|  | Survey person hrs | 3.1 | 9.50 | 0 | 4.5 | 1.0 | 2.5 | 1.5 | 0 | 0 |
|  | No. dead individuals | 0 | 0 | 0 | 0 | 0 | 0 | 0 | 0 | 0 |
|  |  |  |  |  |  |  |  |  |  |  |
|  | No. animals tested | 0 | 2* | 0 | 0 | 0 | 0 | 0 | 0 | 0 |
|  | % positive for *Bd* | - | 50 | - | - | - | - | - | - | - |
|  |  |  |  |  |  |  |  |  |  |  |
| **Area D** | No. obs/survey person hr | 0 | 6.0 | 0.7 | 5.9 | 0 | 0 | 0 | 0.4 | 1.5 |
|  | No. individuals | 0 | 18 | 4 | 68 | 0 | 0 | 0 | 4 | 18 |
|  | No. Post-metamorphosis | 0 | 10 | 4 | 0 | 0 | 0 | 0 | 0 | 0 |
|  | Survey person hrs | 0.5 | 3.0 | 6.0 | 11.5 | 5.2 | 5.4 | 12.8 | 11.4 | 11.7 |
|  | No. dead individuals | 0 | 1.0 | 24.0 | 0 | 0 | 0 | 0 | 0 | 0 |
|  |  |  |  |  |  |  |  |  |  |  |
|  | No. animals tested | 0 | 11* | 0 | 0 | 0 | 0 | 0 | 4 | 13 |
|  | % positive for *Bd* | - | 9 | - | - | - | - | - | 0 | 0 |
|  |  |  |  |  |  |  |  |  |  |  |
| **Area E** | No. obs/survey person hr | 0 | 0 | 0 | 0 | 0 | 0 | 0 | 0 | 0 |
|  | No. individuals | 0 | 0 | 0 | 0 | 0 | 0 | 0 | 0 | 0 |
|  | Survey person hrs | 0.5 | 3.0 | 1.0 | 6.0 | 6.5 | 0.5 | 11.0 | 4.4 | 2.0 |
|  | No. dead individuals | 0 | 0 | 0 | 0 | 0 | 0 | 0 | 0 | 0 |
|  |  |  |  |  |  |  |  |  |  |  |
|  | No. animals tested | 0 | 0 | 0 | 0 | 0 | 0 | 0 | 0 | 0 |
|  | % positive for *Bd* | - | - | - | - | - | - | - | - | - |
|  |  |  |  |  |  |  |  |  |  |  |
| **Area F** | No. obs/survey person hr | 0 | 0 | 0 | 0 | 0 | 0 | 0 | 0 | 0 |
|  | No. individuals | 0 | 0 | 0 | 0 | 0 | 0 | 0 | 0 | 0 |
|  | Survey person hrs | 0 | 0 | 0 | 0.7 | 0.7 | 0.5 | 0.7 | 1.5 | 1.2 |
|  | No. dead individuals | 0 | 0 | 0 | 0 | 0 | 0 | 0 | 0 | 0 |
|  |  |  |  |  |  |  |  |  |  |  |
|  | No. animals tested | 0 | 0 | 0 | 0 | 0 | 0 | 0 | 0 | 0 |
|  | % positive for *Bd* | - | - | - | - | - | - | - | - | - |
|  |  |  |  |  |  |  |  |  |  |  |
| **Area G** | No. obs/survey person hr | 0 | 0 | 0 | 0 | 0 | 0 | 0 | 0 | 0 |
|  | No. individuals | 0 | 0 | 0 | 0 | 0 | 0 | 0 | 0 | 0 |
|  | Survey person hrs | 0 | 0 | 0 | 0 | 0 | 0 | 0 | 0 | 8.3 |
|  | No. dead individuals | 0 | 0 | 0 | 0 | 0 | 0 | 0 | 0 | 0 |
|  |  |  |  |  |  |  |  |  |  |  |
|  | No. animals tested | 0 | 0 | 0 | 0 | 0 | 0 | 0 | 0 | 0 |
|  | % positive for *Bd* | - | - | - | - | - | - | - | - | - |
