# Supplementary material for: Long‐term monitoring of tropical alpine habitat change, Andean anurans, and chytrid fungus in the Cordillera Vilcanota, Peru: Results from a decade of study
Source: Ecol Evol. 2017 Feb 7;7(5):1527–40. doi: 10.1002/ece3.2779 (PMC5330894; doi:10.1002/ece3.2779)
Supplement: Supplementary file 6 [file ECE3-7-1527-s006.docx]

**Supplementary Table S3.** Results of visual encounter surveys for *P. marmoratum* at Areas A-G, and number of individuals tested and prevalence of *Bd* at each location. * Indicates samples that were tested by histology for *Bd*, whereas those without the asterisk were tested by PCR.

|  | ***Pleurodema marmoratum*** | **3-7 March, 2003 (Wet)** | **8-15 August, 2003 (Dry)** | **17-31 July, 2004 (Dry)** | **16-20 March, 2005 (Wet)** | **10-20 March, 2008 (Wet)** | **27 July -6 August, 2009 (Dry)** | **2-13 April, 2012 (Wet)** | **19-26 March, 2013 (Wet)** | **15-25 March, 2015 (Wet)** |
| --- | --- | --- | --- | --- | --- | --- | --- | --- | --- | --- |
| **Area A** | No. obs/survey person hr | 17.0 | 5.4 | 2.0 | 0 | 0 | 1.8 | 0 | 4.2 | 0 |
|  | No. individuals | 17 | 38 | 18 | 0 | 0 | 19 | 0 | 24 | 0 |
|  | No. Post-metamorphosis | 0 | 38 | 18 | 0 | 0 | 19 | 0 | 15 | 0 |
|  | Survey person hrs | 1.0 | 7.0 | 8.8 | 0 | 0 | 10.3 | 0 | 5.75 | 0 |
|  | No. dead individuals | 0 | 0 | 0 | 0 | 0 | 0 | 0 | 0 | 0 |
|  |  |  |  |  |  |  |  |  |  |  |
|  | No. animals tested | 0 | 11 | 0 | 0 | 0 | 0 | 0 | 11 | 0 |
|  | % positive for *Bd* | - | 27.3 | - | - | - | - | - | 45.4 | - |
|  |  |  |  |  |  |  |  |  |  |  |
| **Area B** | No. obs/survey person hr | 0 | 0 | 0 | 6.0 | 27.3 | 0 | 181.8 | 2.0 | 0 |
|  | No. individuals | 0 | 0 | 0 | 1 | 129 | 0 | 409 | 4 | 0 |
|  | No. Post metamorphosis | 0 | 0 | 0 | 1 | 1 | 0 | 15 | 4 | 0 |
|  | Survey person hrs | 0 | 0 | 0.33 | 0.167 | 4.733 | 0 | 2.25 | 2.0 | 0 |
|  | No. dead individuals | 0 | 0 | 1 | 0 | 0 | 0 | 0 | 0 | 0 |
|  |  |  |  |  |  |  |  |  |  |  |
|  | No. animals tested | 0 | 0 | 0 | 0 | 0 | 0 | 10 | 4 | 0 |
|  | % positive for *Bd* | - | - | - | - | - | - | 70 | 75 | - |
|  |  |  |  |  |  |  |  |  |  |  |
| **Area C** | No. obs/survey person hr | 0.96 | 0.2 | 0 | 93.3 | 6.0 | 0.8 | 2.7 | 0 | 0 |
|  | No. individuals | 3 | 2 | 0 | 420 | 6 | 2 | 4 | 0 | 0 |
|  | No. Post-metamorphosis | 3 | 2 | 0 | 30 | 6 | 2 | 4 | 0 | 0 |
|  | Survey person hrs | 3.1 | 9.5 | 0 | 4.5 | 1.0 | 2.5 | 1.5 | 0 | 0 |
|  | No. dead individuals | 0 | 0 | 0 | 1 | 0 | 0 | 1 | 0 | 0 |
|  |  |  |  |  |  |  |  |  |  |  |
|  | No. animals tested | 0 | 0 | 0 | 0 | 0 | 0 | 5 | 0 | 0 |
|  | % positive for *Bd* | - | - | - | - | - | - | 60 | - | - |
|  |  |  |  |  |  |  |  |  |  |  |
| **Area D** | No. obs/survey person hr | 2 | 0 | 0 | 5.3 | 15.9 | 1.8 | 3.6 | 5.5 | 2.7 |
|  | No. individuals | 1 | 0 | 0 | 61 | 82 | 10 | 46 | 62 | 31 |
|  | No. Post-metamorphosis | 0 | 0 | 0 | 3 | 5 | 10 | 28 | 28 | 22 |
|  | Survey person hrs | 0.5 | 3.0 | 6.0 | 11.5 | 5.16 | 5.42 | 12.78 | 11.36 | 11.65 |
|  | No. dead individuals | 0 | 0 | 0 | 0 | 0 | 0 | 0 | 1 | 0 |
|  |  |  |  |  |  |  |  |  |  |  |
|  | No. animals tested | 0 | 0 | 0 | 1 | 0 | 0 | 23 | 21 | 19 |
|  | % positive for *Bd* | - | - | - | 0 | - | - | 52.7 | 28.6 | 10.5 |
|  |  |  |  |  |  |  |  |  |  |  |
| **Area E** | No. obs/survey person hr | 30.0 | 0 | 0 | 337.8 | 16.0 | 2.0 | 37.4 | 48.6 | 19.3 |
|  | No. individuals | 15 | 0 | 0 | 2027 | 104 | 1 | 411 | 215 | 38 |
|  | No. Post-metamorphosis | 0 | 0 | 0 | 5 | 3 | 1 | 18 | 2 | 0 |
|  | Survey person hrs | 0.5 | 3.0 | 1.0 | 6.0 | 6.5 | 0.5 | 11.0 | 4.42 | 1.97 |
|  | No. dead individuals | 0 | 0 | 0 | 0 | 0 | 0 | 0 | 0 | 0 |
|  |  |  |  |  |  |  |  |  |  |  |
|  | No. animals tested | 0 | 0 | 0 | 4 | 0 | 0 | 1 | 2 | 0 |
|  | % positive for *Bd* | - | - | - | 0 | - | - | 100 | 0 | - |
|  |  |  |  |  |  |  |  |  |  |  |
| **Area F** | No. obs/survey person hr | 0 | 0 | 0 | 300 | 0 | 0 | 4.5 | 6.7 | 23.3 |
|  | No. individuals | 0 | 0 | 0 | 201 | 0 | 0 | 3 | 10 | 28 |
|  | No. Post-metamorphosis | 0 | 0 | 0 | 1 | 0 | 0 | 0 | 1 | 0 |
|  | Survey person hrs | 0 | 0 | 0 | 0.67 | 0.67 | 0.50 | 0.67 | 1.5 | 1.2 |
|  | No. dead individuals | 0 | 0 | 0 | 0 | 0 | 0 | 0 | 0 | 0 |
|  |  |  |  |  |  |  |  |  |  |  |
|  | No. animals tested | 0 | 0 | 0 | 2 | 0 | 0 | 0 | 0 | 0 |
|  | % positive for *Bd* | - | - | - | 50 | - | - | - | - | - |
|  |  |  |  |  |  |  |  |  |  |  |
| **Area G** | No. obs/survey person hr | 0 | 0 | 0 | 0 | 0 | 0 | 0 | 0 | 2.1 |
|  | No. individuals | 0 | 0 | 0 | 0 | 0 | 0 | 0 | 0 | 17 |
|  | No. Post-metamorphosis | 0 | 0 | 0 | 0 | 0 | 0 | 0 | 0 | 10 |
|  | Survey person hrs | 0 | 0 | 0 | 0 | 0 | 0 | 0 | 0 | 8.28 |
|  | No. dead individuals | 0 | 0 | 0 | 0 | 0 | 0 | 0 | 0 | 0 |
|  |  |  |  |  |  |  |  |  |  |  |
|  | No. animals tested | 0 | 0 | 0 | 0 | 0 | 0 | 0 | 0 | 10 |
|  | % positive for *Bd* | - | - | - | - | - | - | - | - | 10 |
